# The Intego database: background, methods and basic results of a Flemish general practice-based continuous morbidity registration project

**DOI:** 10.1186/1472-6947-14-48

**Published:** 2014-06-06

**Authors:** Carla Truyers, Geert Goderis, Harrie Dewitte, Marjan vanden Akker, Frank Buntinx

**Affiliations:** 1 Department of general practice, Kapucijnenvoer 33 blok j bus 7001 te B-3000 Leuven, Katholieke Universiteit Leuven, Leuven, Belgium; 2 Department of Family Medicine, Maastricht University, P Debijeplein 1, room A3.014, P.O. Box 616, Maastricht 6200 MD, The Netherlands

**Keywords:** Morbidity registration network, General practice, Methodology, Quality assessment

## Abstract

**Background:**

Intego is the only operational computerized morbidity registration network in Belgium based on general practice data. Intego collects data from over 90 general practitioners. All the information is routinely collected in the electronic health record during daily practice.

**Methods:**

In this article we describe the design and methods used within the Intego network together with some of its basic results. The collected data, the quality control procedures, the ethical-legal aspects and the statistical procedures are discussed.

**Results:**

Intego contains longitudinal information on 285 357 different patients, corresponding to over 2.3% of the Flemish population representative in terms of age and sex. More than 3 million diagnoses, 12 million drug prescriptions and 29 million laboratory tests have been recorded.

**Conclusions:**

Intego enables us to present and compare data on health parameters, incidence and prevalence rates, laboratory results, and prescribed drugs for all relevant subgroups on a routine basis and is unique in Belgium.

## Background

General practioners such as William Pickles in the UK and Frans Huyghen in the Netherlands founded general practice-based epidemiology in the last century. They kept meticulous notes about patients’ disease histories and other relevant information. They realised that general practice provided them with the opportunity to study morbidity in an unselected population over many decades [1].

Computerized patient records in general practice brought a significant change. Databases could be created with longitudinal data covering millions of patient-years. It became easier to study health parameters, diagnoses, laboratory results, prescribed drugs and their interrelations. Many networks were established each with their own accents.

Intego is one of those general practice databases with information from over 90 general practitioners and currently covering 285 thousand individual patients and over two million patient-years. All information is routinely collected and derived from the electronic health record during daily practice. The network was founded in 1994 and data is sent to a central database at regular intervals. Its initial aim was to collect incidence rates of diagnoses presented to the general practitioner. Almost half of the Belgian general practitioners work in solo-practices, without additional staff and rather demand driven. Theoretically all patients can directly contact a specialist, although most patients will be referred to them by a general practitioner [2]. Specialists should inform the general practitioner by means of a digital letter. Patients are not registered in a particular practice, unlike in the Netherlands, where the general practitioner serves as a gatekeeper, but tend to visit the same practice over time.

The gathered information is used as a basis for teaching, quality improvement interventions, and research, as well as for policy making by both physicians’ organisations and governmental bodies. Over the years, the network’s aims broadened and now also include the natural history of diseases, etiological and prognostic research, evaluation of population-based interventions with respect to prevention and quality improvement and relations between diagnostic categories, laboratory results and prescribed drugs. The network is mainly funded by the Health ministry of the Flemish government. Ad hoc research projects serve as an additional funding source.

In this paper we describe the design and methods used within the Intego network together with some of its basic results.

## Methods

### Data collection

At the start of the Intego-project it was decided to use the medical electronic health record - Medidoc® ^-^ which is one of the few existing packages in Belgium allowing routine input of coded diagnoses and other coded data. All data are recorded by the general practitioner using keywords in a predetermined field in the electronic health record. Each of the 67500 keywords is associated with a unique program-specific internal code and can be linked to classifications as ICPC-2, ICD-10 and ATC-DDD (Table 1).

**Table 1:** Notation of diagnoses in Medidoc

**Free text**	**Keyword**	**Program code**	**ICPC-code**	**ICD-9-CM** **code**	**ICD-10 code**
Complex fracture of leg			→ L73		
	Fracture tibia diaphysis closed	→ Q82320			
		Q82320	→ L73	→ 823.20	→ S82.20

When a general practitioner agrees to participate a software module copies the data from the general practitioner into a text file. For apparent privacy reasons each patient is assigned a random number. This file is then encrypted and sent to an independent trusted third party that recodes the data and subsequently sends it to the department of general practice of the KU Leuven where it is stored into the central database. The trusted third party procedure has been in place since 2012.

### Quality of the data

#### Selection procedure

General practitioners willing to participate in Intego have to pass three general quality criteria. First, the average number of new diagnoses per patient per year should be higher than one. This number was chosen based on a graphical presentation of the average number of new dagnoses per year, which showed a clear cutpoint at one. This number corresponds with what was found in the Nijmegen Continuous Morbidity Registration [2]. Secondly, the percentage of diagnoses recorded without using keywords should be less than five percent. Finally, these parameters must remain stable for at least three years. With these criteria, in particular the first one, we intend to minimize the risk of recording bias, where general practitioners only register certain, e.g. serious, diagnoses.

### Collected data

The following data are copied from the general practitioner’s electronic health record into the Intego database: year of birth, gender, the years that the patient contacted the practice, diagnoses, drug prescriptions, laboratory results, some biomedical parameters such as blood pressure, height, weight, smoking status and mortality. Other lifestyle information, such as nutrition, is not recorded.

Once the data are in the database a check is performed to see whether the data are as correct and complete as possible [3-5]. With regard to registration networks, correctness refers to whether a patient with a diagnosis in the electronic health record also suffers from that particular disease. Completeness refers to whether the record of a patient with a certain disease contains that diagnosis. Validation of a network can be performed by means of external (comparison with other registries or studies as a measure of completeness) and internal methods (tracers as a measure of correctness). Tracers are known relationships between background characteristics, gender, age, laboratory test results or medication and disease: e.g. patients receiving insulin treatment should also have a registered diagnosis for diabetes.

External validation of the Intego database has been examined by means of national and international comparisons. Nationally, overall cancer incidence was compared to the Limburg Cancer Registry (LIKAR). Our incidence rates of influenza and acute respiratory illness were compared to the European Influenza Surveillance Scheme, EISN [6]. Data found in Intego are routinely compared to the Tweede nationale studie, studying morbidity and care in Dutch general practice [7]. Serious infections in children were compared to data from other networks [8]. Several diseases were compared to the Dutch CMR (Nijmegen) and RNH (Maastricht).

Other validation methods are either irrelevant in these systems (for example comparison with paperbased data) or impossible (for example merging with other databases such as cancer registries) because patients cannot be identified due to privacy reasons. The feasibility of linking patient data from other sources (e.g., hospital) with Intego data by means of a trusted third party is currently under study.

### Analysis method

#### Basic analysis

Intego can provide data about incidences and prevalences of diseases, distribution of risk factors, time trends, age- and gender differences and drug use.

Incidence is calculated as the number of new cases of disease divided by the person-time magnitude [9]. Incidence rates can be provided for the total population, or stratified by age group and/or gender or some other ad hoc defined subgroup and can be standardized for the Flemish, Belgian, European or World population. The denominator is the yearly contact group (YCG), patients which visit the practice at least once in a given year or the practice population, all patients in a practice, consisting of the YCG plus the group which does not visit their general practitioner in a given period. This last group is estimated from all non-profit health insurance agencies, which includes complete data on all reimbursed medical and paramedical acts, age, gender and residence as well as the total insured population. A yearly correction factor is calculated, to estimate the percentage of patients consulting a general practitioner by age group, gender and region and hence the practice population.

The prevalence of a population is the proportion of the population with the disease at a specified time. Unlike incidence rates, which focus on new events, prevalence focuses on existing states. Because of the design of Intego (no episode registration and no recording of cure) prevalence rates can only be calculated on incurable chronic diseases, such as diabetes.

Gender differences are usually expressed as sex ratios, which represent the incidences in female to male rate (or vice versa). Age is divided into Wonca-age groups or analysed as a continuous variable.

Identifying rising and declining trends in disease and other medical data is an important part of epidemiology. Environmental changes, prevention, public health action, behavioral changes etc. can all result in disease incidence changes. The Intego database can provide data from 1994 until now and can study time trends or seasonal effects in diseases using standard time series analysis techniques [10], [6].

Medication is coded according to the Belgian coding system CNK (national codes) which can easily be translated into the ATC-DDD format. These data can be used for pharmacovigilance and pharmacoepidemiological purposes.

### Design

Many study designs can be used, such as incidence studies, retrospective cohort studies, case‒control studies and cross‒sectional designs in an unselected patient group or subgroup. For each individual study, the sample population is chosen according to specific inclusion and exclusion criteria depending on the aim of the study.

### Statistics and data analysis

When a research question is formulated, and the use of morbidity registration data is deemed relevant to answer this question, a protocol is written, data is collected, statistically analyzed and results are summarized. There are many approaches for making inferences from observational studies, some focusing on the data collection (design), others on the statistical analyses. However, even with the best designs, observational studies do not automatically control for selection bias. Therefore matching, standardization, stratification and/or covariance adjustment are needed [11]. The purpose of these techniques is to create index and control groups eliminating bias as much as possible. Matching and covariate adjustment are most often used. For covariate adjustment the variables that should be balanced (mostly at least age, gender and practice) are put explicitly in the statistical model and this way adjusted for. This method could however produce biased results if there is an additional imbalance in background variables which are not stated in the model (e.g. smoking or other confounders). Matching is based on at least gender, age (+/- 5 years) and within practices and constitutes most likely of a 5 to 1 ratio [12]. Standardization can be performed with any relevant population, including one of the comparison groups. Most frequently used are the standard Flemish or European populations. The latter is most popular in cancer related epidemiological studies [13]. Statistical techniques which are often used are ordinary least squares regression, logistical regression, and survival analysis.

An internal manual for analyzing repeatedly introduced data, such as laboratory (e.g. HbA1c) or clinical data (e.g. blood pressure) was constructed in order to standardize analysis procedures disposable for all researchers using the database. For instance, in some longitudinal analyses, we aggregate individual values on a yearly basis. For LDL-cholesterol and HbA1c, we take the last value of each year, but for creatinine e.g. we take the average value of the last two measurements of each year in order to account for the important within subject variability of this variable [14].

### Legal ethical aspects

At the start of the project a proposal was submitted to the Belgian Privacy Commission. No comments were provided by them. The procedures were also reviewed and approved by the ethical review board of the medical school of the Katholieke Universiteit Leuven [15]. More recently, the Sectoral Committee ‘Health’ of the Belgian privacy commission thoroughly reviewed our procedures and asked for the use of a third trusted party for additional recoding of the original coded data and for the installation of a scientific and ethical review board. These boards consist of researchers, both from our department and from other universities, Intego registering practitioners, a non-medical reviewer and an ethicist and lawyer. They assess whether the questions are ethically correct (Ethics Board) and scientifically sound (Scientific Board). They have an advisory function, comparable to a Data Safety Monitoring Board in clinical trials. After these improvements our procedures are compliant with the law confirmed by decision nr. 13.026 of March 19^th^, 2013.

## Results

### General practitioners

Between 1999 and 2012, 144 different practices provided their data. Based on the criteria specified above, 51 practices (representing 95 general practitioners) were selected for inclusion in the database. The practices are spread throughout Flanders, but are not selected to be representative for the Flemish general practitioners. The general practitioners in the project represent slightly fewer women and are slightly older than the Flemish general practitioners overall.

### Patients

**Figure 1:**
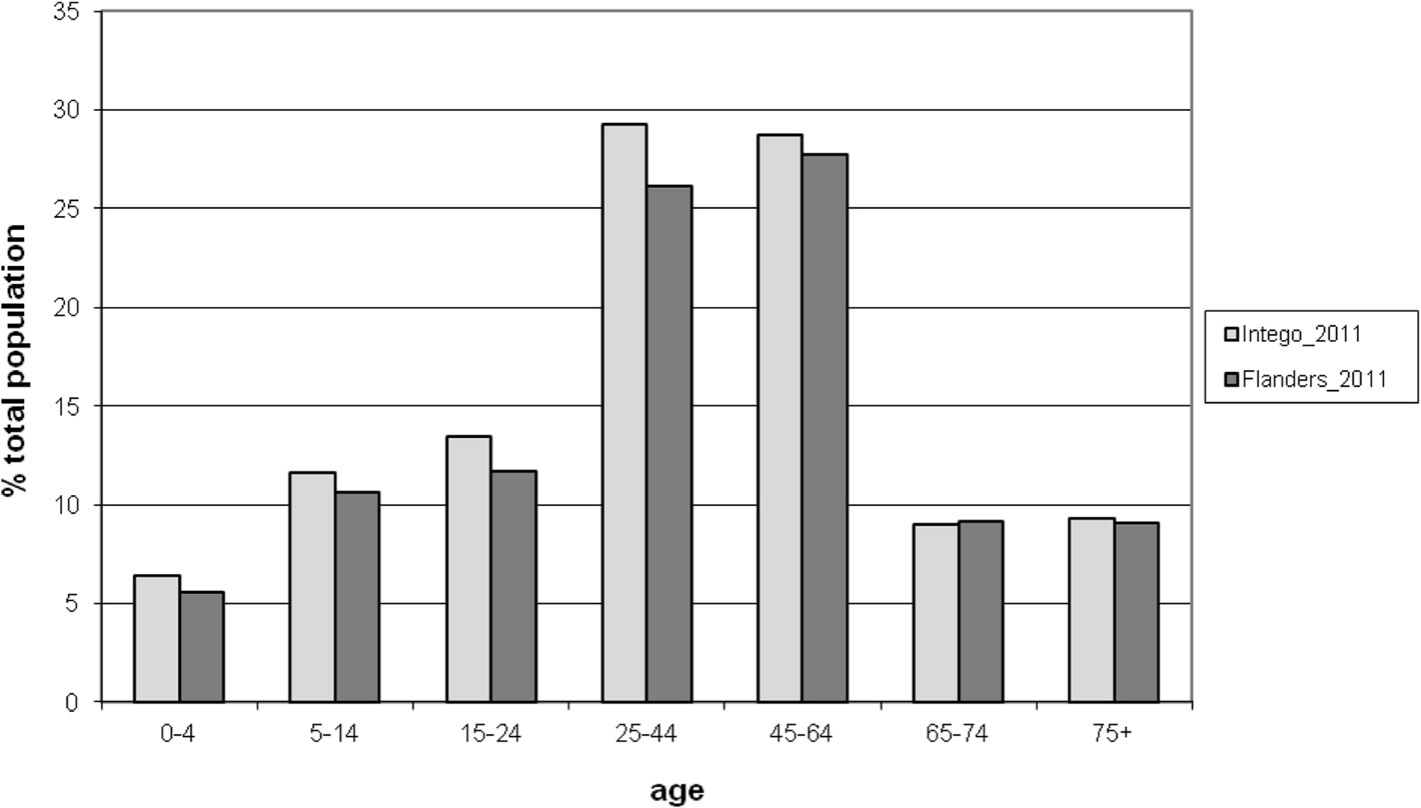
The Flemish population and the population of the Intego-database by age.

The database contains information on 285 357 different patients, with 2 million patient-years for 1994–2011. In the year 2011, 115 303 of these patients contacted their general practitioner. This corresponds to an estimated practice population of 143 495, which is 2.3% of the Flemish population. The Intego-population is representative for the Flemish population in terms of age and sex (Figure 1). The largest difference is 3.2% in the age group 25–44 years. In all these patients 3 million diagnoses, 12 million drug prescriptions and 29 million laboratory tests have been recorded.

### Data

**Figure 2:**
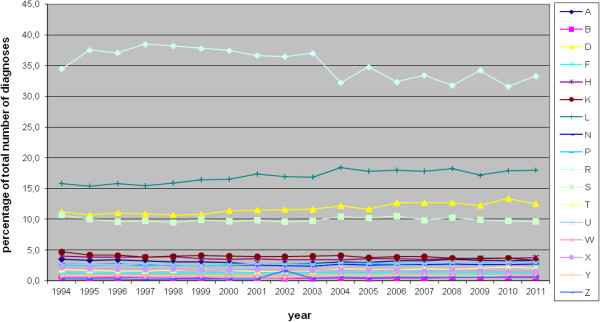
Proportion of new diagnoses per ICPC-chapter.

The proportion of new diagnoses in each ICPC-chapter is stable over the years (Figure 2) with respiratory disorders making up the vast majority of diagnoses (33% of all new diagnoses in 2011). These are followed by musculoskeletal disorders with 18%, gastro-intestinal disorders with 13% and skin diseases with 10%. These four chapters together constitute 74% of all new diagnoses. The 13 remaining chapters each represent less than 4% of all new diagnoses. These proportions correspond well to the 20 most common diagnoses (Table 2), with infections of the respiratory tract and diseases of the locomotor apparatus at the top of the list.

**Table 2:** The 20 most frequent new diagnoses in 2011

**Female**				**Male**			
**ICPC Code**	**Diagnosis**	**Total number**	**‰**	**ICPC Code**	**Diagnosis**	**Total number**	**‰**
R74	Upper respiratory infection acute	40391	233	R74	Upper respiratory infection acute	36248	226
R80	Influenza	13016	75	R80	Influenza	13151	82
D73	Gastroenteritis presumed infection	12305	71	D73	Gastroenteritis presumed infection	12060	75
R78	Acute bronchitis/bronchiolitis	10288	59	R78	Acute bronchitis/bronchiolitis	10230	64
U71	Cystitis/urinary infection other	8080	47	L03	Low back symptom/complaint	7345	46
L03	Low back symptom/complaint	7427	43	L87	Bursitis/tendinitis/synovitis nos	5583	35
L83	Neck syndrome	6987	40	S16	Bruise/contusion	4702	29
R75	Sinusitis acute/chronic	6454	37	H71	Acute otitis media/myringitis	4409	28
L87	Bursitis/tendinitis/synovitis nos	5992	35	L83	Neck syndrome	4148	26
H71	Acute otitis media/myringitis	4434	26	R75	Sinusitis acute/chronic	3699	23
R76	Tonsillitis acute	4225	24	R76	Tonsillitis acute	3647	23
S16	Bruise/contusion	4166	24	R77	Laryngitis/tracheitis acute	2803	17
D87	Stomach function disorder	3987	23	S74	Dermatophytosis	2615	16
R77	Laryngitis/tracheitis acute	3786	22	D87	Stomach function disorder	2547	16
L92	Shoulder syndrome	3002	17	L92	Shoulder syndrome	2519	16
R05	Cough	2526	15	R05	Cough	2242	14
L86	Back syndrome with radiating pain	2450	14	D84	Oesophagus disease	2053	13
D84	Oesophagus disease	2423	14	L86	Back syndrome with radiating pain	2050	13
A04	Weakness/tiredness general	2373	14	K86	Hypertension uncomplicated	1873	12
S74	Dermatophytosis	2183	13	S18	Laceration/cut	1762	11

A typical analysis dataset contains event dates for all variables under study and patient characteristics (e.g. gender and year of birth).

Basic incidence rates are calculated by 1000 or 100,000 person-years and stratified by gender. For example the incidence rate of malignant melonoma is 8/100,000 patient-years overall, and 6.5 and 9.7/100,000 patient-years for males and females. In many cases results are also presented by age groups. Prevalence rates can be calculated for chronic conditions.

Because all prescriptions are registered in the electronic health record these data can be used for studies on drug prescriptions (e.g. the relationship between atypical antipsychotic use and subsequent risk of diabetes) [16].

Several international peer reviewed articles have been published using data from the Intego database. For example, Intego produced antibiotic prescribing indicator values. These values reveal huge opportunities to improve the quality of antibiotic prescribing, especially the prescription of recommended antibiotics [17]. Next to this, it was found that chronic kidney disease is highly prevalent in patients with type 2 diabetes mellitus and that only a minority of patients evolve into severe decline which is associated with younger age, male gender, a year-to-year decline of >10 mL/min and manageable factors such as blood pressure, blood glucose, associated drugs prescriptions including statin therapy [14]. Another example is low back pain (LBP). Most studies on comorbidity in LBP have been conducted in specialized settings with the use of self-reports. Low back pain is one of the most frequent diagnoses in general practice. Striking is the higher frequency of common self-limiting diseases in patients with a diagnosis of LBP during the same year [18]. In another study a moderate significant association between herpes zoster and subsequent cancer risk in women older than 65 years was found, without any influence of antiviral therapy. No association was found with herpes simplex [19].

### Quality of the data

We continuously compare our results with results from other registries and groups. Up to now, there is no gold standard to measure the quality of registered data. Registering practitioners have to pass the above mentioned quality criteria. It is however not clear to what extent these criteria really increase the quality of gathered data. It is also unclear whether supplemental criteria can be useful to increase the quality. External validation of the data with other registries compares similar databases which may suffer from the same biases. A comparison with registers such as the NIVEL primary care database or IPCI database in the Netherlands or the Health Improvement Network (THIN) database in the UK might be interesting for this purpose, since they use both coded and free text data. All are large longitudinal general practice research databases which are based on routinely collected data of a non selected population. Comparison to data from databases where all general practitioners who want to participate can participate in Intego which selects their general practitioners based on stringent registration quality criteria might provide useful insights to our quality criteria.

With regard to cancer incidences, Intego and LIKAR respectively registered 3.83 and 3.63 cases of invasive cancers (exclusion of basal cell carcinoma) per 1,000 patient years [20]. Incidences of melanoma (8 versus 8.55 /100,000 patient years), lung (12 and 79 versus 15 and 79/100,000 patient years for females and males) and cervical cancer (9 versus 9.13 /100,000 patient years) were also very comparable [21,22]. Comparisons on transient ischemic attacks (TIA) and stroke were performed with the general practice sentinel network (Belgian Scientific Institute for Public Health), resulting in comparable incidences for stroke and higher incidences for TIA in Intego (1.93 versus 1.73 for stroke and 0.89 versus 1.29 /1,000 patient years for TIA) [23]. In preparation of the H_1_N_1_ vaccination campaign the incidence of Guillain Barré was examined by comparing the number registered in the database for the most recent years and numbers based on recollection by the general practitioners, assuming every general practitioner remembers the cases in his/her practice because of its low incidence and its dramatic clinical picture. Both were in very high agreement, meaning around 1/100,000 patient years and higher than a specialist (neurologists) registration. Comparison with the EISN showed high agreement with regard to Influenza like illness and acute respiratory infections [6].

The yearly incidence of serious infections in children aged 0 – 4 years in the Continuous Morbidity Registration (CMR) is 27–30/1,000 patients, 18.3 in the Dutch Transition project, 18.1 in Intego and 15.7 in the Weekly Returns Service (the latter is based on children from 1–4 years old) [8]. Several comparisons with CMR and RNH resulted in similarities and differences but for none of the networks a specific direction was found with regard to the differences. The eHID project, with the objective to investigate the operational features of networks providing epidemiological information based on the extraction of routinely collected health related data in order to make recommendations on best recording practices, compared disease frequency in several European member states. The Intego data were comparable to other networks [24].

Internal validation can be performed using tracers. For diabetes and anti-diabetics 88% of the people who were prescribed anti-diabetics (ATC, A10A, A10B and A10X) also had a diabetes diagnosis. With regard to dementia (ATC, N06DA), migraine (ATC, N02CC) and Parkinson (ATC, N04A and N04B), associations were lower, respectively 52%, 69% and 36%. Quality improvement projects are currently ongoing to study these results.

## Discussion

### Strenghts

Computerisation provides relatively inexpensive and easy access to large volumes of data. They are population based and can quickly produce large samples of patients. Because of the longitudinal nature of the data, many research questions can be answered in a more time and cost effective manner compared to other study designs (e.g. newly set up cohort studies).

The most frequently used design is the retrospective cohort design which enables researchers to analyze a large amount of data collected and carefully stored in the past, while analyzing them using a cohort design. The design enables researchers to use information collected in tempero non suspecto, without any hindsight of the objectives of the actual study and avoiding the main risks of bias in prospective study designs, especially selection and recall bias. Thus, the retrospective design guarantees that the measurement of predictor variables was not biased by knowledge of which subjects had the outcome of interest. In a prospective design, knowledge of exposure status may bias classification of the outcome. Also, in a prospective design, being in the study may alter participant’s behavior. Since data in a registry are routinely collected, this inclusion bias does not count for retrospective study designs. Compared to a case–control design, an advantage of the retrospective cohort design is that all of the subjects who developed the outcome (cases) and all those who did not (control) come from the same population.

A major strength of these types of cohort studies in general is the possibility to study multiple exposures and multiple outcomes in one cohort. Even rare exposures can be studied. The combined effect of multiple exposures on disease risk can be determined. Hypothesis generation is another benefit of cohort studies considered as a way to pick up associations between many exposures and outcomes. Yet because of lack of randomization, cohort studies do not permit conclusions about causality. Several underlying etiological hypotheses can be generated, to be tested in other confirmatory studies.

Deckers et al. formulated minimal criteria for a primary care network [25]. Computerisation provides relatively inexpensive and easy access to large volumes of data. A sufficient sample size is advised to be about 1% of the population, which allows the study of common diseases. Increasing the sample size much more will lead to an increased workload, but is not expected to result in additional information. Intego covers more than 2% of the Flemish population, highly representative for age and gender. Data on age is collected on a continuous scale and can be divided into as many age groups as needed. Data from the previous registration year are collected in the first half of the year. Although data are not collected on a weekly basis, they can be reconstructed in retrospect to obtain weekly, monthly or even daily information.

Because Intego is based on routinely collected data and the study population is selected with broader inclusion and less exclusion criteria compared to an RCT, its results may be more generalizable to clinical practice.

### Weaknesses

Some variables, which may be important confounders in public health research are not measured (occupation, employment, and socioeconomic status), measured imprecisely, or even unknown, such as smoking or mortality, which is only registered partially. Information from specialists as well as events that occur in hospital may not be fully captured in the electronic health record. Over the counter medications and treatments given in hospital are not readily available. Exposure status (e.g. onset of treatment, exact year of diagnosis) may be missed because it has occurred prior to start of the registration.

The most ill can die, others can be lost to follow-up because they moved. This will bias the results when selective follow-up rates differ between index and reference group. Loss to follow-up can be related to future outcome. The Healthy Survivor Effect (HSE) can be described as a continuing selection process in the cohort due to survival or maintenance of the healthiest individuals, whereas survival/maintenance process may differ amongst the selected groups (e.g. diabetes or not). The study will include only patients remaining in the system, a survivor (healthier) population. HSE can lead to lower than expected outcomes, can interact with exposure vs. outcome associations between groups (e.g. statin effect in longitudinal design on elderly diabetic patients with and without CKD). When there is no exposure-disease relationship, higher cumulative exposure appears protective of health. The effects of healthy patient effect biases may vary by gender, race/ethnicity, social class, work status, age at inclusion, length of follow-up or cause of mortality/morbidity.

Differential misclassification can lead to an overestimation or underestimation of the effect between exposure and outcome. Classification of individuals (exposure or outcome status) can also be affected by changes in diagnostic procedures.

Intego is based on routinely collected data. Does the general practitioner make a correct diagnosis or assessment? This refers to the discussion about diagnosis (cough, cold,…) in primary care, but also to the problem of episode registration and changing diagnoses (ex. Mycoplasma pn. Infection). Criteria based diagnoses are more accurate than symptom based diagnoses. Second, has the diagnosis/assessment been correctly coded in the electronic health record? This question refers to the sensitivity (or completeness) and the positive predictive value (or correctness) of electronic health record -based data [26].

Although the patient population is representative for the Flemish population, registering general practitioners are not representative for the general practictioner population. It is a selected group of high quality registering practitioners which use a specific electronic health record. This selection bias of general practitioners could eventually have an influence on some process parameters in the follow-up of patients.

Difficulties arise with tracers, when some drugs are prescribed for other conditions as well. For example anti-epileptics are also prescribed for chronic pain. Also a general practitioner might have a very specific reason for describing a specific drug to a specific person. The lower associations between dementia, migraine and Parkinson with their tracer medications probably were caused by this lack of specificity.

### Future

In the near future, large databases with all kinds of information will be widely available. Some of these registries will contain data of almost the entire population of a country or a region. This is a challenging situation for the existing databases collecting data from a smaller sample population. We will have to consider redefining our strategic goals, methods and procedures.

The option of collecting either blood or buccal smear samples of patients in Intego is being explored [27]. Together with background characteristics and a full inventory of recorded morbidity, laboratory results and prescribed drugs, this would enable research on gene-environment and pharmaco-genetic interactions. This can only be performed after strict informed consent and with the agreement of the ethical review board, the national privacy authorities, and the Flemish authorities supervising the Flemish Biobank activities.

We will continue to explicitly structure quality procedures with training and coaching, introduce criteria for diagnosis (for example GOLD criteria for COPD classification) and list missing data for possible confounders, such as smoking and SES. We will check the validity of our 3 existing quality criteria and continue to work out and implement internal and external validation procedures. We are convinced that this procedure will procure a quality label enabling small registries like Intego to occur as a gold standard for large databases with a lot of data often extracted from ‘bad’ registrators.

Finally, registries like Intego with a stable group of practicising general practitioners also offers possibilities for targeted, experimental, randomized trials.

## Conclusion

With 95 general practitioners and data covering over two million patient-years, Intego certainly is not one of the largest general practice based computerized morbidity registries in the world. Nevertheless, the database enables us to present and compare data on background information, incidence and prevalence rates, laboratory results, and prescribed drugs for all relevant subgroups on a routine basis and is unique in Belgium. This results from two basic requirements: a group of dedicated general practitioners keeping high quality patient records using highly structured software and a procedure that does not require them to do anything special, additional to this record keeping.

## Competing interests

The authors declare that they have no competing interests.

## Authors’ contributions

All authors participated in the design of the study and helped to draft the manuscript. All authors read and approved the final manuscript.

## Pre-publication history

The pre-publication history for this paper can be accessed here:

http://www.biomedcentral.com/1472-6947/14/48/prepub
